# Intermediate multidomain state in single-crystalline Mn-doped BiFeO_3_ thin films during ferroelectric polarization switching

**DOI:** 10.1038/s41598-024-65215-w

**Published:** 2024-06-21

**Authors:** Seiji Nakashima, Koji Kimura, Naohisa Happo, Artoni Kevin R. Ang, Yuta Yamamoto, Halubai Sekhar, Ai I. Osaka, Koichi Hayashi, Hironori Fujisawa

**Affiliations:** 1 Department of Electronics and Computer Science, Graduate School of Engineering, University of Hyogo, Himeji, Hyogo 671-2201 Japan; 2 Department of Physical Science and Engineering, Nagoya Institute of Technology, Nagoya, 466-8555 Japan; 3 Research Center for Advanced Measurement and Characterization, National Institute for Materials Science, 1-2-1 Sengen, Tsukuba, Ibaraki 305-0047 Japan; 4 Japan Synchrotron Radiation Research Institute, Super Photon Ring-8GeV (SPring-8), Sayo, 679-5198 Japan; 5 Department of Computer and Network Engineering, Graduate School of Information Sciences, Hiroshima City University, Asa-Minami-Ku, Hiroshima, 731-3194 Japan; 6 Toyota Technological Institute, Nagoya, Aichi 468-8511 Japan; 7 Graduate School of Science and Technology, Nara Institute of Science and Technology, Ikoma, 630-0192 Japan

**Keywords:** Ferroelectrics and multiferroics, Actuators

## Abstract

A intermediate multidomain state and large crystallographic tilting of 1.78° for the (*hh*0)_pc_ planes of a (001)_pc_-oriented single-domain Mn-doped BiFeO_3_ (BFMO) thin film were found when an electric field was applied along the [110]_pc_ direction. The anomalous crystallographic tilting was caused by ferroelastic domain switching of the 109° domain switching. In addition, ferroelastic domain switching occurred via an intermediate multidomain state. To investigate these switching dynamics under an electric field, we used in situ fluorescent X-ray induced Kossel line pattern measurements with synchrotron radiation. In addition, in situ inverse X-ray fluorescence holography (XFH) experiments revealed that atomic displacement occurred under an applied electric field. We attributed the atomic displacement to crystallographic tilting induced by a converse piezoelectric effect. Our findings provide important insights for the design of piezoelectric and ferroelectric materials and devices.

## Introduction

Ferroelectric semiconductors, in which charge carriers can be controlled through spontaneous polarization and interband transitions of the excited carriers, have attracted much attention for use in novel AI electronic devices^1–4^ or photovoltaic devices^4–10^ that exceed the Shockley–Queisser limit^11^. The photovoltaic effect in ferroelectrics with a noncentrosymmetric crystal structure has been well investigated as a bulk photovoltaic effect (BPVE) that induces optical strains^11–15^ by coupling with a converse piezoelectric effect. More recently, ultra-fast optical-induced strains and polarization modulations in (110)-oriented BiFeO_3_ single-domain film by femto-second laser irradiation^16^. Thus, the characteristics and lattice distortions of ferroelectric semiconductors are deeply coupled via spontaneous polarization (*P*_s_), indicating that ferroelastic lattice distortion with ***P***_**s**_ vector rotation causes drastic modulation of the characteristics of ferroelectric semiconductors. Therefore, clarifying ferroelectric switching dynamics from the viewpoint of electric-field-induced strain is becoming increasingly important.

The electric-field-induced strains in Pb(Zr,Ti)O_3_^17–19^ with a morphotropic phase boundary (MPB) composition or relaxer ferroelectrics^20^ have been well investigated, and non-180° domain switching with ferroelastic lattice distortion is well known to induce a large converse piezoelectric response. In addition, in La-doped BFO, epitaxial growth at low temperature of 450 °C which is CMOS process compatible have been reported^21^, indicating the BFO-based materials are expected for novel device application. More recently, in (001)_pc_-oriented single-domain BiFeO_3_ (BFO)^22^ nanoislands, 71° domain switching has been induced before 180° domain switching^23^, meaning that the activation energy for the former is lower in a strain-free BFO crystal. Indeed, we have already reported that epitaxial Mn-1-at%-doped BFO (BFMO) thin films on the twin boundary of a bicrystal SrTiO_3_ substrate shows 71° ferroelastic domain switching^24^. In thin films, domain switching process should be more complex because of existence of lattice clamping at ferroelectrics/substrate interface. The domain switching in ferroelectric thin films have been well investigated by applying electric fields along thickness direction. However, there are few reports^25,26^ of applying electric field along in-plane direction, despites importance for the device application.

Here, we conducted an in situ investigation of ferroelectric domain switching in (001)_pc_-oriented single-crystalline BFMO thin films under an electric field applied along the in-plane direction and found an anomalous ferroelastic lattice tilting of ~ 1.78° during ferroelectric domain switching via intermediate multidomain state. Moreover, we conducted in situ atomic-scale observations of the thin films under an applied electric field and found that the *A*-site ions are dislocated. Fluorescent X-ray induced Kossel line pattern measurement and inverse X-ray fluorescence holography (XFH) technique with synchrotron radiation were used for the in situ observations. XFH is a recently developed technique for acquiring crystallographic and local atomic structural information with element selectivity^27–33^. In the present paper, we demonstrate in situ XFH measurements under an applied electric field.

## Results

### Intermediate multidomain state and crystallographic tilting of single-domain BFMO thin films under applied electric field

**Figure 1:**
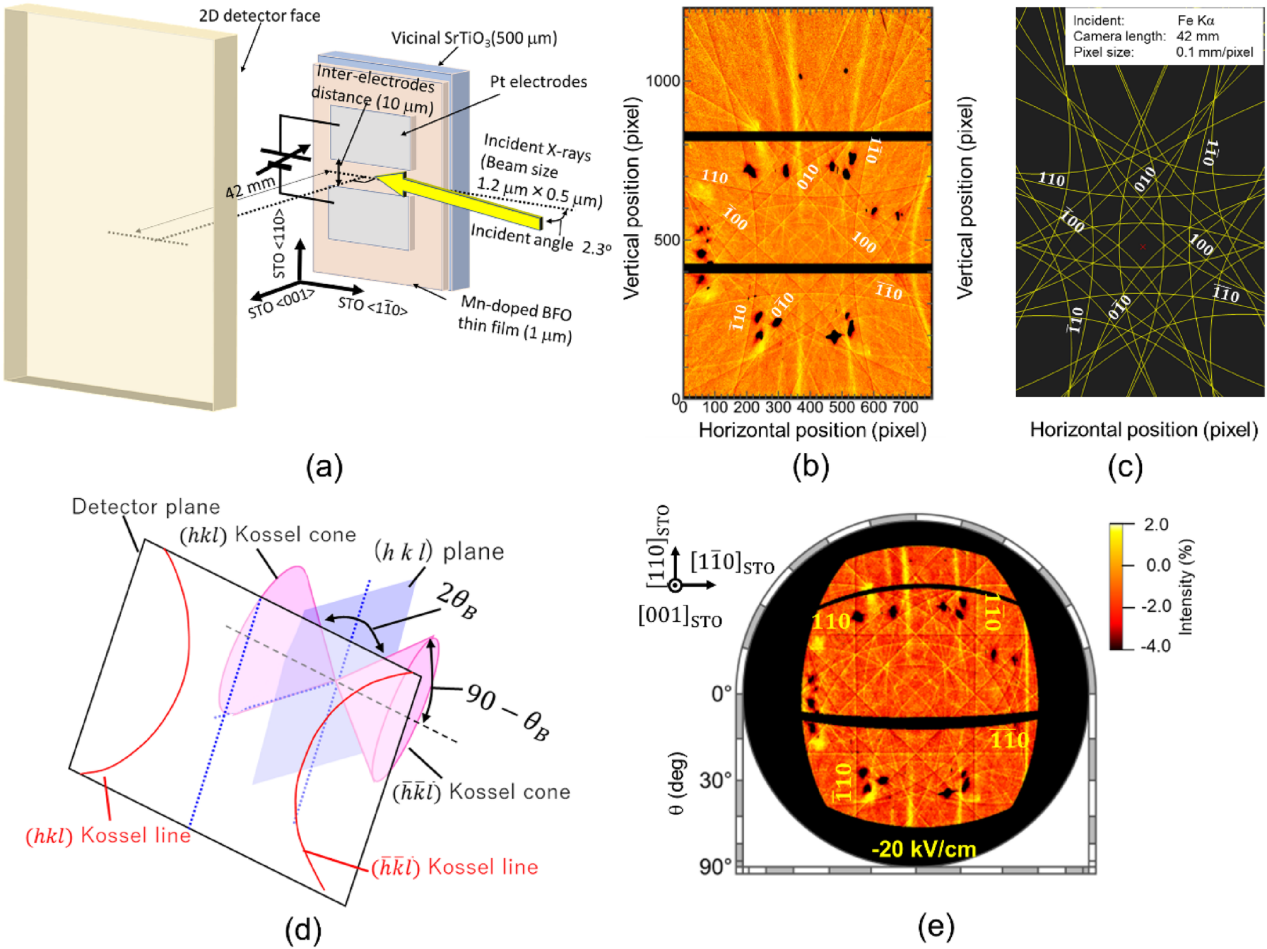
In situ Kossel line pattern measurements under electric field. (**a**) Schematic of sample structure and measurement setup. (**b**) Fe Kα Kossel line pattern under − 20 kV/cm applied electric field after poling by application of − 200 kV/cm electric field. (**c**) Calculated Kossel pattern by Fe Kα radiation. (**d**) Schematic of Kossel pattern detection by 2D detector. (**e**) Orthogonal projection along [001]_pc_ direction of Fe Kα Kossel line pattern under − 20 kV/cm applied electric field, projected onto Ewald sphere in *k*-space.

Investigating the ferroelectric switching dynamics of BFMO thin films requires crystallographic characterization under an electric field. For this purpose, we conducted in situ normal XFH measurements under applied electric fields of various strengths. Schematics of the fabricated Pt/BFMO/Pt coplanar capacitor structure and measurement setup are shown in Fig. 1a. The 1-μm-thick BFMO thin films have been grown on the vicinal SrTiO_3_ (001) substrate by RF planar magnetron sputtering. As shown in Fig. S1a in Supplementary materials, surface AFM image indicates that the 1-μm-thick BFMO thin films shows well-aligned step-and-terrace structure. Uniform contrast of the vertical- and lateral-PFM images, and XRD reciprocal space mapping results reveals the BFMO thin films were single-domain (See Figs. S1b,c and S2 in Supplementary materials). The crystallographic and ferroelectric properties of a 1-μm-thick BFMO thin film on a vicinal SrTiO_3_(001) substrate have been reported elsewhere^10^. In the present study, 40-nm-thick Pt electrode patterns with a width of 700 μm were fabricated on a 1-μm-thick single-crystalline BFMO thin film/vicinal SrTiO_3_(001) (STO) single-crystal heterostructure. The Pt electrodes were aligned along the [110]_STO_ direction and the inter-electrode distance was set to 10 μm. (See Fig. S3a in supplementary materials) When a voltage was applied between these Pt electrodes, an electric field was applied to the BFMO thin films along the [110]_pc_ direction. Synchrotron X-ray radiation with an energy of 7.30 keV was focused with a beam size of 1.2 × 0.5 μm^2^ onto the area between these Pt electrodes. After careful background subtraction, geometric curves pattern called Kossel lines pattern can be obtained. Figure 1b shows the Fe Kα Kossel lines pattern obtained under a − 20 kV/cm applied field after poling was carried out by applying a − 200 kV/cm field; the black spots in the patterns are masked area of strong and sharp diffraction peaks of incident X-rays, because such so strong diffraction peaks hinder background removal. In a normal XFH measurement, excited fluorescence X-rays are diffracted by arbitrary (*hkl*) planes under the Bragg reflection condition. The Fe Kα Kossel pattern agreed well with the calculated pattern by using Recipro software^34^, as shown in Fig. 1c, and can be assigned to the crystallographic planes. The diffracted X-rays with Bragg reflection conditions form a cone shape called a Kossel cone (Fig. 1d). The Kossel cone was cut by the 2D detector plane, resulting in circular, elliptical, hyperbolic, or parabolic shapes of the Kossel line patterns. The Fe Kα Kossel line pattern was projected onto an Ewald sphere in *k*-space and an orthographic projection image was obtained (Fig. 1e). In this orthographic projection image, the Kossel lines that originated from the crystallographic planes perpendicular to the projection plane should be straight lines, indicating clear ${110}_{\text{pc}}$, ${\overline{1 }\overline{1}0 }_{\text{pc}}$, ${1\overline{1}0 }_{\text{pc}}$, and ${\overline{1}10 }_{\text{pc}}$ Kossel lines. Notably, dark lines are emphasized in the ${110}_{\text{pc}}$ Kossel pattern, whereas bright lines are emphasized in the ${\overline{1 }\overline{1}0 }_{\text{pc}}$ pattern, which is attributed to the noncentrosymmetric crystal structure of BFMO (rhombohedral system with space group *R*3*c*).

**Figure 2:**
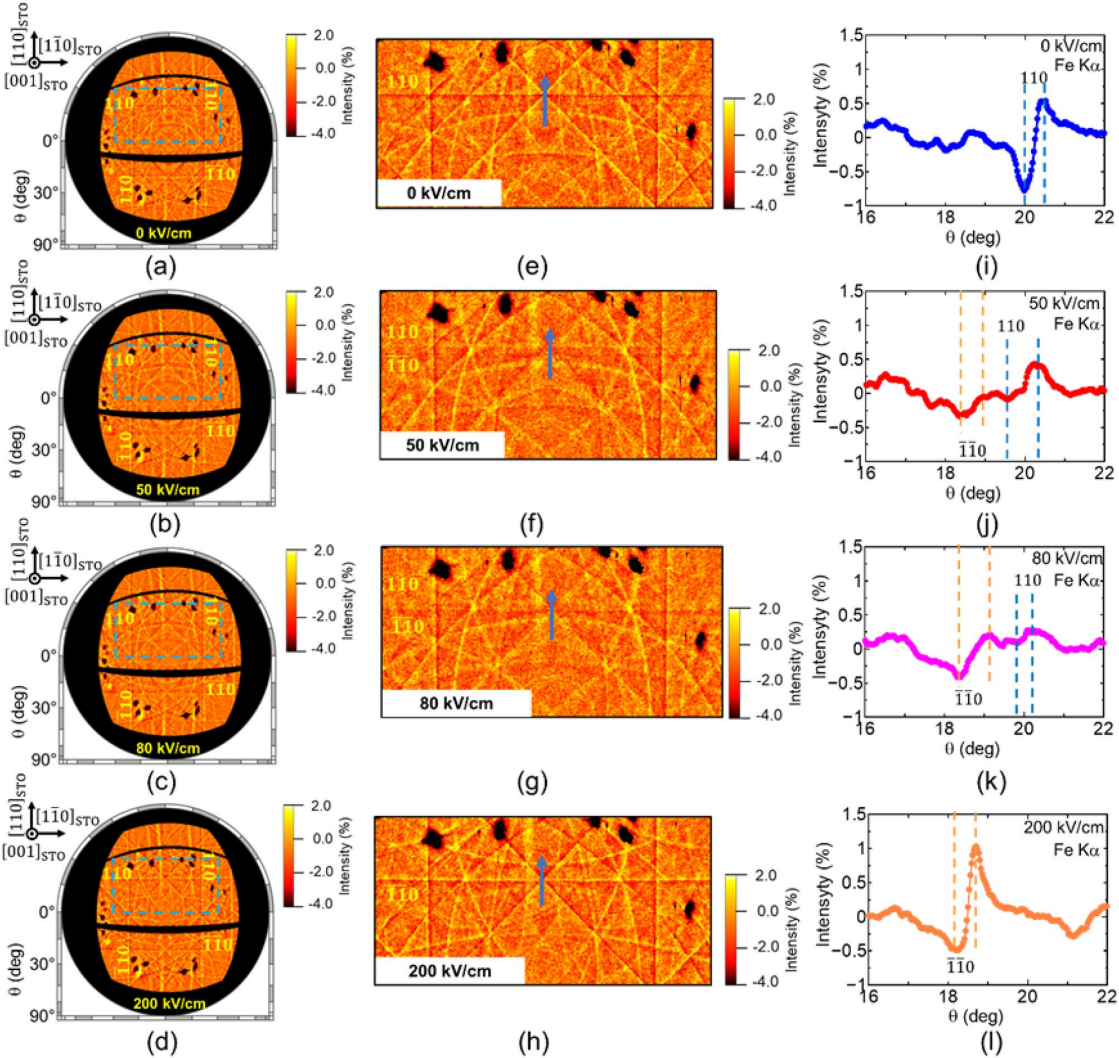
Fe Kα Kossel line patterns under electric field of 0–200 kV/cm. (**a**–**d**) Orthographic projection of Fe Kα Kossel line pattern projected onto Ewald sphere in *k*-space. (**e**–**h**) Magnified images of regions indicated by blue-dashed lines in (**a**–**d**), respectively. (**i**–**l**) Cross-sectional profiles along blue arrows in (**e**–**h**), respectively, under (**a**, **e**, **i**) 0 kV/cm, (**b**, **f**, **j**) 50 kV/cm, (**c**, **g**, **k**) 80 kV/cm, and (**d**, **h**, **l**) 200 kV/cm fields. Under 50 and 80 kV/cm fields, the BFMO thin film shows an intermediate multidomain state because of anomalous domain switching.

To investigate the ferroelectric domain switching dynamics, we increased the electric field from 0 to 200 kV/cm to induce ferroelectric polarization switching. Figure 2a–d show Fe Kα Kossel line patterns acquired under applied electric fields of 0, 50, 80, and 200 kV/cm, respectively. With increasing electric field intensity, the Fe Kα Kossel line pattern clearly changed, indicating that the electric field induced crystallographic changes. To investigate these changes in detail, we acquired magnified images of the areas indicated by blue dashed lines in Fig. 2a–d (Fig. 2e–h, respectively). Moreover, cross-sectional profiles along the blue arrows in Fig. 2e–h are shown in Fig. 2i–l, respectively. Although a clear ${110}_{\text{pc}}$ Kossel line with dark line enhancement was detected under 0 kV/cm (Fig. 2e,i), a clear ${\overline{1 }\overline{1}0 }_{\text{pc}}$ Kossel line with bright line enhancement was detected under 200 kV/cm (Fig. 2h,l). In a single-domain thin film, the ${110}_{\text{pc}}$ and ${\overline{1 }\overline{1}0 }_{\text{pc}}$ planes are parallel; however, these ${110}_{\text{pc}}$ and ${\overline{1 }\overline{1}0 }_{\text{pc}}$ Kossel lines were detected at different *θ* positions, indicating non-180° domain switching. After domain switching, the *hh*0 planes are tilted 1.78° along the ${\left[\overline{1 }\overline{1 }0\right]}_{\text{STO}}$ direction. By contrast, under 50 and 80 kV/cm applied fields, both ${110}_{\text{pc}}$ and ${\overline{1 }\overline{1}0 }_{\text{pc}}$ Kossel lines are detected simultaneously (Fig. 2f,g,j,k), indicating a multidomain state. The angles between the ${110}_{\text{pc}}$ and ${\overline{1 }\overline{1}0 }_{\text{pc}}$ planes under 50 and 80 kV/cm applied fields are estimated to be approximately the same: 1.30°.

**Figure 3:**
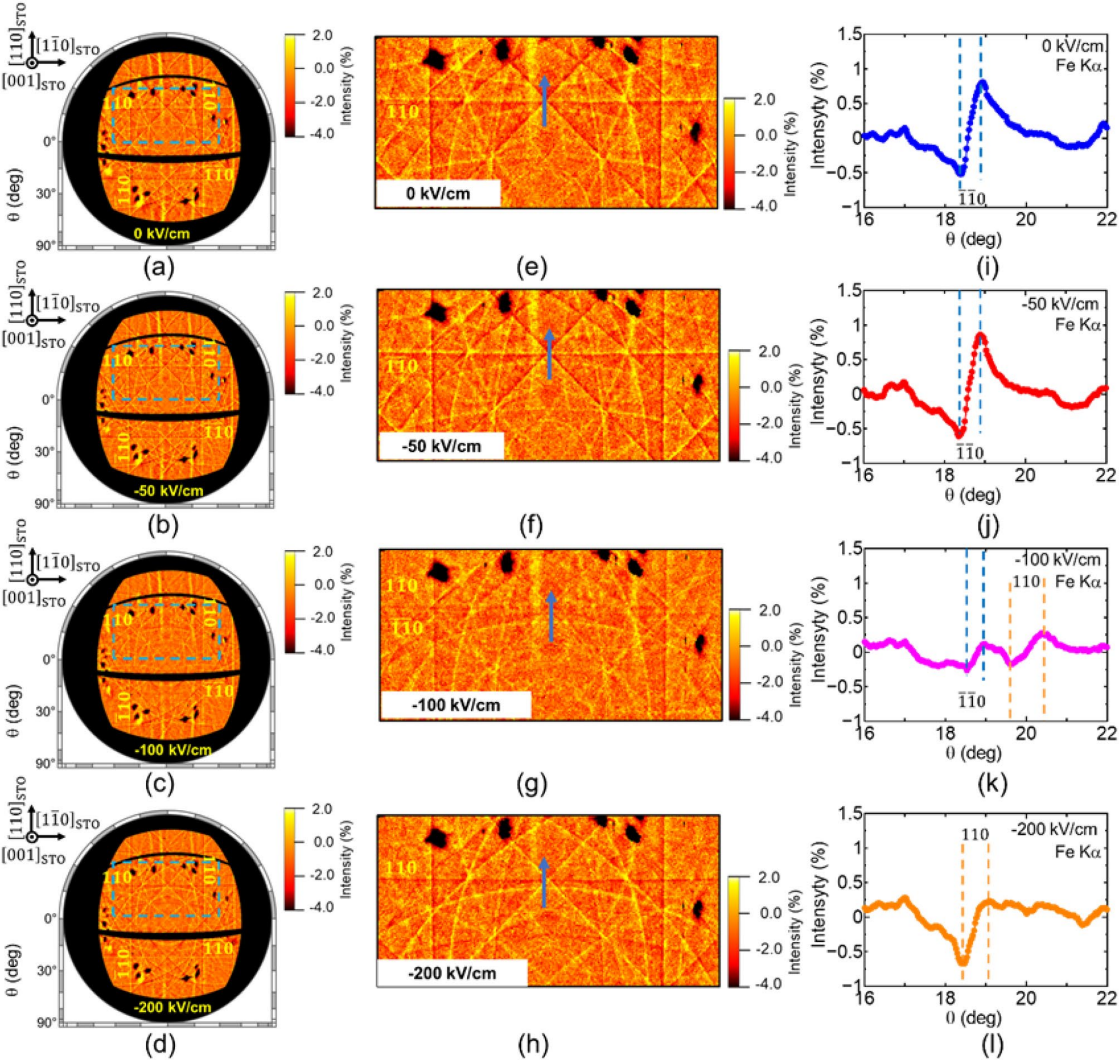
Fe Kα Kossel line patterns under electric field of 0–200 kV/cm. (**a**–**d**) Orthographic projection of Fe Kα Kossel line pattern projected onto Ewald sphere in *k*-space. (**e**–**h**) magnified images of regions indicated by blue-dashed lines in (**a**–**d**), respectively. (**i**–**l**) Cross-sectional profiles along blue arrows in (**e**–**h**), respectively, under (**a**, **e**, **i**) 0 kV/cm, (**b**, **f**, **j**) − 50 kV/cm, (**c**, **g**, **k**) − 100 kV/cm, and (**d**, **h**, **l**) − 200 kV/cm electric fields. Under a − 100 kV/cm field, the BFMO thin film shows an intermediate multidomain state because of anomalous domain switching.

After a 200 kV/cm electric field was applied, the electric field was decreased from 0 to − 200 kV/cm to induce switching in the reverse direction. Figure 3a–d show Fe Kα Kossel line patterns under electric fields of 0, − 50, − 100, and − 200 kV/cm, respectively. With decreasing electric field strength, the Fe Kα Kossel line pattern shows a clearly different tendency compared with the case shown in Fig. 2. For a detailed investigation, we acquired magnified images of the areas indicated by blue dashed lines in Fig. 3a–d (Fig. 3e–h, respectively). Moreover, cross-sectional profiles along the blue arrows in Fig. 3e–h are shown in Fig. 3i–l, respectively. Under 0 and − 50 kV/cm fields, ${\overline{1 }\overline{1}0 }_{\text{pc}}$ Kossel lines are detected at approximately the same *θ* position (Fig. 3e,f,i,j). In addition, the ${110}_{\text{pc}}$ Kossel line under a − 200 kV/cm field is detected at approximately the same *θ*_B_ position (Fig. 3g,k), indicating polarization switching without *hh*0 plane tilting. By contrast, under a − 100 kV/cm field corresponding to an intermediate state, both the ${110}_{\text{pc}}$ and ${\overline{1 }\overline{1}0 }_{\text{pc}}$ Kossel lines are detected simultaneously at different *θ* positions (Figs. 2k and 3g), indicating a multidomain state. In the intermediate state, the angle between the ${110}_{\text{pc}}$ and ${\overline{1 }\overline{1}0 }_{\text{pc}}$ planes can be estimated to be 1.30°, which is the same state achieved under 50 and 80 kV/cm applied fields (Fig. 2i,j). Thus, although the final states of polarization switching in Figs. 2 and 3 differ from each other, polarization switching occurs via the same intermediate state.

### Local atomic structure of single-domain BFMO thin films under electric field

The most effective method to clarify the ferroelectric domain switching dynamics is to measure atomic structures under applied electric fields. For such a challenging study, we used inverse XFH measurements, which are based on the time-reversal phenomenon of normal XFH. Although the detailed theory of inverse XFH has been described elsewhere^27–30^, the most important feature is that atomic images can be reconstructed because multiple hologram patterns can be acquired by incidence of X-rays with multiple energies.

**Figure 4:**
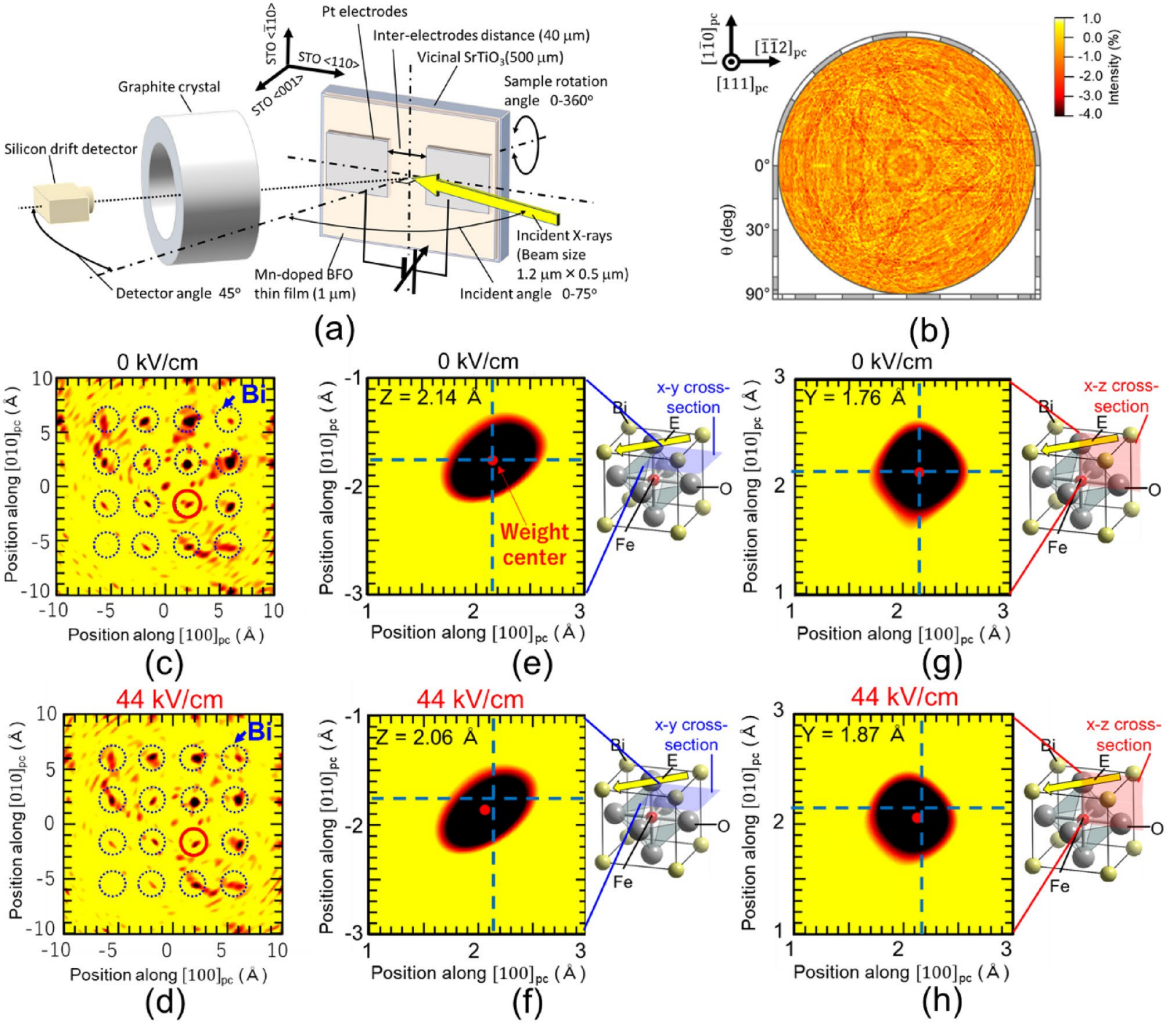
Atomic structure measurements under 0 and 44 kV/cm by in situ inverse XFH. (**a**) Schematic of sample structure and measurement setup for inverse XFH. (**b**) Orthographic projection of Fe Kα hologram pattern acquired under 0 kV/cm, projected onto Ewald sphere in *k*-space after symmetric operations belonging to point group 3 m, (**c**, **d**) Two-dimensional atomic images at *z* = 2 Å plane. (**e**, **f**) *x*–*y* and (**g**, **h**) *x*–*z* cross-sectional images around Bi atoms indicated by red circles in (**c**, **d**), respectively, under (**c**, **e**, **g**) 0 kV/cm and (**d**, **f**, **h**) 44 kV/cm. The red dots in (**e–h**) are weight centers of Bi atoms, showing displacement under electric field. For the {*x*, *y*, *z*} coordinate system, the *x*–*y* and *x*–*z* planes are defined as the [*h*00]_pc_–[0*k*0]_pc_ and [*h*00]_pc_–[00* l*]_pc_ planes, respectively, and the origin of (*x*, *y*, *z*) = (0, 0, 0) is set at the position of the Fe atom.

Schematics of the measurement setup and sample structure are shown in Fig. 4a. For reconstruction of the atomic structure by inverse XFH measurements, the sample should rotate to enable detection of wide-area hologram patterns. Thus, we increased the distance between the Pt electrodes on BFMO thin films to 40 μm to prevent X-ray irradiation of areas where no electric field is applied even if the rotational axis is eccentric. (See Fig. S3b in supplementary materials) We acquired an Fe Kα hologram pattern by varying the X-ray incidence angle *θ* from 0° to 75° and the sample rotation angle *ϕ* from 0° to 360°. The measurement was repeated with the incident X-ray energy varied from 7.5 to 11.0 keV in 0.5 keV steps, resulting in eight Fe Kα hologram patterns. Moreover, these measurements were performed under 0 and 175 V (44 kV/cm) potentials applied between the Pt electrodes. To expand the hologram patterns and improve the signal-to-noise (S/N) ratio, symmetric operations of three-fold rotation along [111]_pc_ and mirror operation for the ${\left[\overline{1 }10\right]}_{\text{pc}}$ plane belonging to point group 3* m* were applied to these Fe Kα hologram patterns, resulting in an Fe Kα hologram pattern (Fig. 4b). This pattern is an orthogonal projection along the [111]_pc_ direction, acquired at an incident X-ray energy and an applied electric field of 11.0 keV and 0 kV/cm, respectively. Clear X-ray standing-wave lines with threefold symmetry were confirmed. Although the hologram patterns do not cover the whole $4\uppi$ sr sphere face, meaning that deformation of the atomic structure cannot be completely ignored, the atomic structure under 0 and 44 kV/cm applied fields can be relatively compared.

By Fourier transforming these holograms using Barton's multi-wavenumber-transformation algorithm^35^, the atomic structure around the Fe atom under 0 and 44 kV/cm applied fields can be reconstructed (Fig. 4c–h). Atomic images in the *z* = 2 Å plane under 0 and 44 kV/cm applied fields are shown in Fig. 4c,d, respectively, where the *z* = 0 Å plane is defined as the (00*l*)_pc_ plane including Fe atoms, meaning that the *z* = 2 Å plane represents the (00*l*)_pc_ plane including Bi atoms. Notably, Bi atoms in the BFMO crystal under 0 and 44 kV/cm applied fields can be reconstructed from in situ inverse XFH measurements conducted under an applied electric field. The atomic images indicate that the Fe atoms occupy the *B* site in the *AB*O_3_-type perovskite structure and that the BFMO thin film is single-crystalline. For a detailed investigation, magnified cross-sectional images of the Bi atoms indicated by red circles in Fig. 4c,d are shown in Fig. 4e–h. The red dots in these figures show weight centers of the Bi atoms under 0 and 44 kV/cm applied fields. The weight center in both the *x*–*y* and the *x*–*z* cross-sections are displaced when the electric field is applied, revealing that Bi atoms are displaced along the ${\left[\overline{1 }\overline{1 }\overline{1 }\right]}_{\text{pc}}$ direction by 0.05 Å under the applied field. This displacement means that the Bi ions (*A*-site ions) exhibit larger displacements than the Fe ions (*B*-site ions) under an electric field. Greater *A*-site ion displacements from a centrosymmetric position have been reported in PbTiO_3_^36^ or BiFeO_3_^37^, in which the *A*-site ions have a stereochemically active lone pair, consistent with the larger Bi-ion displacement in the BFMO thin film under an electric field.

In addition, these displacements under an electric field include a crystallographic tilting component during polarization switching. As previously mentioned, the BFMO thin film showed a multidomain state under a 50 kV/cm applied field (Fig. 2b), revealing that a 44 kV/cm electric field did not reach the threshold electric field for switching to the multidomain state. In addition, crystallographic tilting occurred because of pinning of the lattice shrinkage by electric-field-induced strain at the BFMO/STO interface. Therefore, although we cannot distinguish pure atomic displacement and crystallographic tilting from the atomic image changes induced by an electric field, the displacements of Bi ions along the ${\left[\overline{1 }\overline{1 }\overline{1 }\right]}_{\text{pc}}$ direction agree well, even if both the polarization and lattice shrinking due to the electric-field-induced strains are considered.

## Discussion

**Figure 5:**
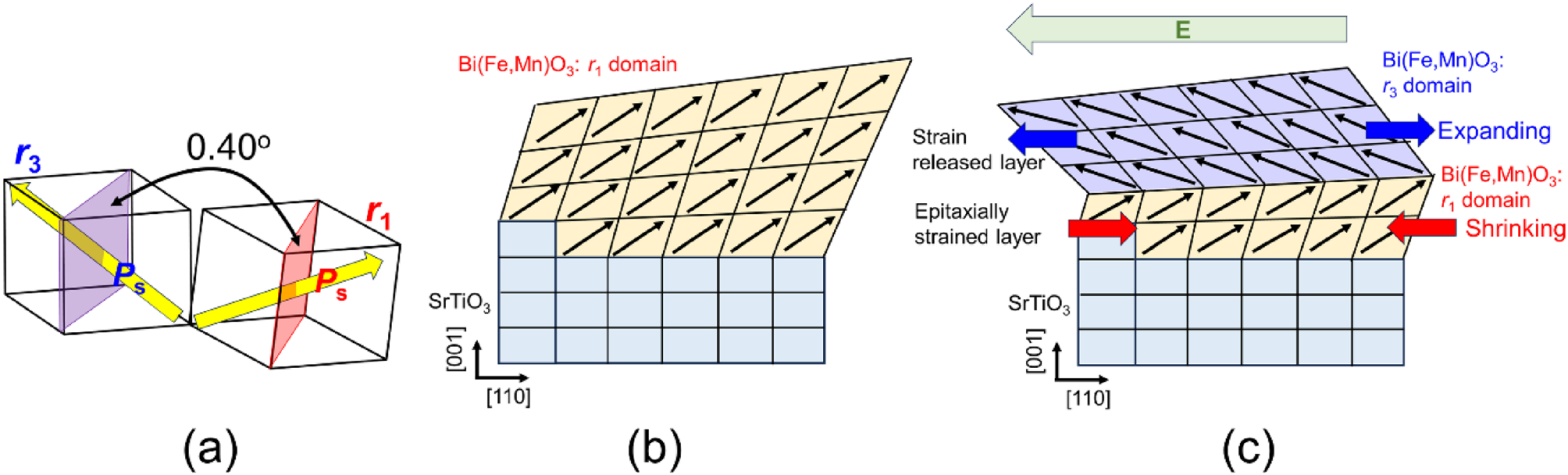
Schematics of ferroelastic 109° domain switching via intermediate multidomain state. (**a**) schematic of pure 109° domain, (**b**) Single-domain BFMO thin film and (**c**) intermediate multidomain structure under electric field. In the 109° domain, the angle between (*hh*0)_pc_ planes in the *r*_1_ and* r*_3_ domain is 0.40°. However, in an epitaxially strained single-domain BFMO film, the epitaxially strained layer cannot easily undergo ferroelastic domain switching, resulting in intermediate multidomain state.

In a (001)_pc_-oriented single-domain BFMO thin film, ferroelectric polarization switching occurred when an electric field was applied along the [110]_pc_ direction (Figs. 1 and 2). Interestingly, You et al. have reported that in-plane polarization switching in a Pt/multidomain BFO/Pt coplanar capacitor showed 71° domain switching after an electric field was applied along the [100]_pc_ direction. However, Fig. 1 reveals that 109° domain switching occurs even in a single-crystalline BFMO thin film via an intermediate multidomain state when an electric field is applied along the [110]_pc_ direction (Fig. 2j,k). Vertical- and lateral-PFM images measured after electric field application also reveal the 109° domain switching (see Fig. S1 in Supplementary materials). For the pure 109° domain switching (Fig. 5a), the angle between the (*hh*0) planes of the *r*_1_ and *r*_3_ domains can be estimated to be 0.40° using previously reported lattice constants for a bulk BFO. By contrast, interestingly, the angle between the (*hh*0)_pc_ planes in the *r*_1_ and *r*_3_ domains is 1.30° in the intermediate multidomain state. Moreover, the angle between the (*hh*0)_pc_ planes under 0 and 200 kV/cm applied electric fields reached 1.78°, which is substantially larger than the bulk BFO case. X-ray diffraction (XRD) reciprocal space mapping around BFMO 004_pc_ and 114_pc_ diffraction spots, as shown in Fig. S2 in Supplementary materials, reveals that the angle between [001]_pc_ direction and (*hh*0)_pc_ plane can be estimated to be 0.63°, meaning the angle between (*hh*0)_pc_ planes of *r*_1_ and *r*_3_ domains should be 1.26°. This value agreed well with the angle between (*hh*0)_pc_ planes of *r*_1_ and *r*_3_ domains in intermediate multidomain state of 1.30°. We speculate that the anomalous lattice tilting originates from lattice pinning at the STO substrate (Fig. 5b). With increasing electric field strength, the strain-released layer can easily cause 109° domain switching, although epitaxially strained layers do not cause such switching. Thus, the lattice in the *r*_1_ domain shrinks, whereas that in the *r*_3_ domain expands, resulting in an anomalous intermediate multidomain state (Fig. 5c).

For the switching reversal process in which the electric field is decreased to − 200 kV/cm (Fig. 3), the same intermediate multidomain state (*r*_1_ and *r*_3_ domains) is produced under a − 100 kV/cm applied field, indicating 109° domain switching. However, in the final state under a − 200 kV/cm field, only the *r*_3_ domain is produced, indicating 180° domain switching. The reason for the reversal of the ferroelastic domain switching remains unknown. However, 109° domain switching with anomalous lattice tilting also occurs during 180° domain switching.

In conclusion, single-domain BFMO thin films show ferroelastic 109° domain switching via an intermediate multidomain state when an in-plane electric field is applied along the [110]_pc_ direction. The angle between (*hh*0)_pc_ planes in the *r*_1_ and *r*_3_ domains in the intermediate 109° multidomain state can be estimated to be 1.30°. Moreover, the angle between the (*hh*0)_pc_ planes in the *r*_1_ domain under a 0 kV/cm field and the *r*_3_ domain under a 200 kV/cm field reached 1.78°. These values are surprisingly large compared with the angle of 0.40° in the case of a pure 109° domain. The anomalous intermediate domain state is attributable to lattice pinning at the BFMO/STO interface. In addition, in the reverse switching process under a negative applied electric field, the same intermediate multidomain state is generated under a − 100 kV/cm field. However, in the final state under a − 200 kV/cm field, the 109° domain vanishes and changes to 180° domain switching, meaning that the domain structure does not return to its original configuration. The reason for this behavior remains unclear; however, we speculate that epitaxial strain and lattice pinning are involved. In addition, the local atomic structure under a 44 kV/cm field reveals that Bi atom displacements of ~ 0.05 Å occur along the ${\left[\overline{1 }\overline{1 }\overline{1 }\right]}_{\text{pc}}$ direction because of the crystallographic tilting induced by the converse piezoelectric response, consistent with the anomalous switching process. These results demonstrating large crystallographic tilting via intermediate multidomain state due to ferroelastic domain switching in single-domain BFMO thin films provide useful insights for piezoelectric materials design and piezoelectric device applications.

## Methods

### Fluorescent X-ray induced Kossel line pattern measurements

Fluorescent X-ray induced Kossel line pattern measurements were conducted to detect interference patterns with fluorescence X-rays from target atoms and fluorescence X-ray scattering at surrounding atoms. For exciting Fe Kα (6.34 keV) fluorescence X-rays, 7.30 keV X-rays were used because of the Fe K-edge at 7.11 keV. The incidence angle of the focused X-rays was kept at 2.3° to prevent excitation of fluorescence from Ti, which is a constituent of the SrTiO_3_ substrate. The Fe Kα fluorescent X-rays from the BFMO thin film were detected using a 2D detector (Rigaku, Hypix-9000) positioned 42 mm from the sample face. An Fe Kα Kossel line pattern image was obtained by integrating for 90 s and repeating the process 11 times under dc electric field application, meaning that it takes for 990 s. During the measurement, dc electric field is applied continuously. The Fe Kα Kossel line pattern was obtained by adding these 11 images following background subtraction. This process was repeated for each electric field application. The measurements were performed at the BL37XU and BL39XU beamlines at the SPring-8 synchrotron radiation facilities, Japan. Hologram data processing involving subtracting the background and converting the data to an orthographic projection was performed using the 3D-AIR-IMAGE holography analysis software^38^.

### Inverse XFH measurements

Inverse XFH is a measurement that detects fluorescence X-ray holograms excited by the interference between incident X-rays and X-rays scattered by surrounding atoms, meaning that hologram patterns excited by X-rays with multiple energies can be detected. To detect the incident X-ray angle dependence of fluorescence X-ray intensity, the X-ray incidence angle *θ* was varied from 0° to 75° in 1° steps and the sample rotation angle *ϕ* was varied from 0° to 360° in 0.25° steps. The Fe Kα fluorescence X-rays excited by incident X-rays were spectralized and focused by a toroidal graphite crystal and detected by a silicon drift detector (SDD). During the measurements, the detector angle was maintained at 45°. The incident X-ray energy was varied from 7.5 to 11.0 keV in 0.5 keV steps, resulting in eight holograms. It takes 24 h for measuring the eight holograms. During the measurements, dc electric field was kept applied. These measurements were performed on the BL13XU and BL37XU beamlines at SPring-8, Japan. Hologram data processing involving subtracting the background, performing symmetry operations, and reconstructing atomic structures was performed using the 3D-AIR-IMAGE software^38^.

### Sample preparation

A 1-μm-thick BFMO thin film was grown on a vicinal STO(001) substrate of which (001) plane is inclined for 4° along <$\overline{1 }\overline{1 }0$>_STO_ direction by a radiofrequency (RF) planar magnetron sputtering process for single domain film growth^39–42^. During growth, the substrate temperature, RF power, sputtering pressure, and Ar/O_2_ gas flow rate were maintained at 650 °C, 35 W, 0.5 Pa, and 3.5 sccm/1.5 sccm, respectively. Mixed and calcined powders of α-Fe_2_O_3_, Bi_2_O_3_, and Mn_2_O_3_ powders with an atomic composition ratio of Bi:Fe:Mn = 1.10:0.99:0.01 were pressed into a disk shape with a diameter of 4 inches and used as a target. The Pt electrode patterns (Figs. 1a and 4a) were fabricated on the BFMO thin films via a photolithography lift-off process using RF magnetron sputtering for Pt deposition at room temperature. The sample photographs for the normal and inverse XFH measurements were shown in Fig. S3a,b, respectively. For applying electric field, Au wires are bonded by silver paste onto the Pt electrodes.

### Supplementary Information

Supplementary Information.

## Data Availability

The datasets analyzed during the current study are available from the corresponding author upon reasonable request.
