# Supplementary material for: Intermediate multidomain state in single-crystalline Mn-doped BiFeO3 thin films during ferroelectric polarization switching
Source: Sci Rep. 2024 Jun 21;14:14358. doi: 10.1038/s41598-024-65215-w (PMC11192801; doi:10.1038/s41598-024-65215-w)
Supplement: Supplementary file 1 — Supplementary Information. [file 41598_2024_65215_MOESM1_ESM.pdf]

Supplementary Materials

Intermediate Multidomain State in Single-crystalline Mn-doped $BiFeO_3$ Thin Films during Ferroelectric Polarization Switching

Seiji Nakashima[1,*], Koji Kimura[2,3,4], Naohisa Happo[5], Artoni Kevin R. Ang[6], Yuta Yamamoto[7], Halubai Sekhar[2,4], Ai Isohashi Osaka[1], Koichi Hayashi[2,4], and Hironori Fujisawa[1]

[1] *Department of Electronics and Computer Science, Graduate School of Engineering, University of Hyogo, Himeji, Hyogo 671-2201, Japan*

[2] *Department of Physical Science and Engineering, Nagoya Institute of Technology, Nagoya 466-8555, Japan*

[3] *Research Center for Advanced Measurement and Characterization, National Institute for Materials Science, 1-2-1 Sengen, Tsukuba, Ibaraki, 305-0047, Japan*

[4] *Japan Synchrotron Radiation Research Institute, Super Photon Ring-8GeV (SPring-8), Sayo, 679-5198. Japan.*

[5] *Department of Computer and Network Engineering, Graduate School of Information Sciences, Hiroshima City University, Asa-Minami-ku, Hiroshima 731-3194, Japan*

[6] *Toyota Technological Institute, Nagoya, Aichi 468-8511, Japan*

[7] *Graduate School of Science and Technology, Nara Institute of Science and Technology, Ikoma 630-0192, Japan*

.

[*] nakashima@eng.u-hyogo.ac.jp

Surface morphology and domain structure of 1-μm-thick Mn-doped $BiFeO_3$ thin film

Figures S1(a)-S1(c) shows AFM, vertical- and lateral-PFM images of as-deposited 1-μm-thick Mn-doped $BiFeO_3$ (BFOM) thin film. Mn doping amount is fixed at 1 at%. The AFM image reveals that the BFMO thin films has step-and-terrace structure with a step propagation direction along $[110]_{STO}$ direction. The step propagation direction corresponds to vicinal direction of the STO substrate. The vertical- and lateral-PFM images show bright and dark uniform contrast, showing phases of the piezoresponse oscillation

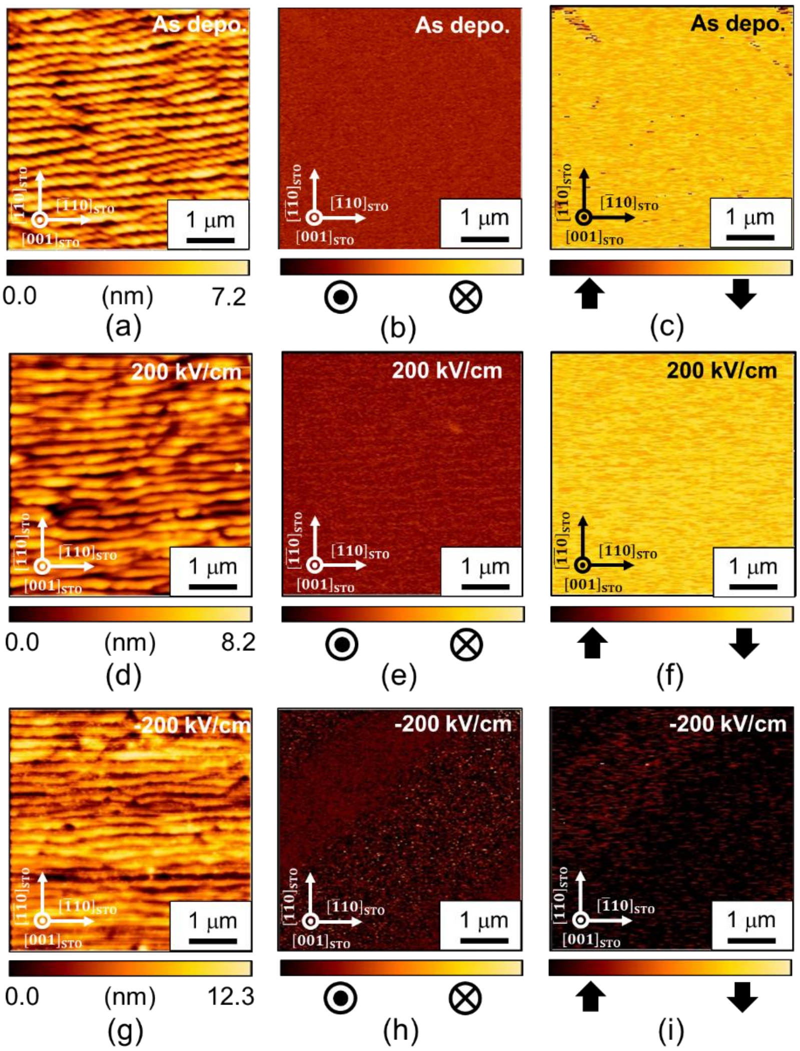

Figure S1 (a), (d), (g) Surface AFM, (b), (e), (h) vertical-PFM, and (c), (f), (i) lateral-PFM images of (a), (b), (c) as-deposited BFMO film, (d), (e), (f) after 200 kV/cm and (g), (h), (i), after -200 kV/cm application.

along vertical and lateral directions aligned in entire region. Therefore, the BFMO thin film has single domain structure. The $\boldsymbol{P}_s$ vector is aligned along $[111]_{pc}$ direction.

Figures S1(d)-S1(f) shows AFM, vertical- and lateral-PFM images of the BFMO thin film after 200 kV/cm application along $[110]_{STO}$ direction. The contrast of vertical- and lateral-PFM image does not change, indicating that the $\boldsymbol{P}_s$ vector is aligned along $[111]_{pc}$ direction.

AFM, vertical- and lateral-PFM images of the BFMO thin film after -200 kV/ cm application are shown in Figs. S1(g)-S1(i). Only contrast of the lateral-PFM image is reversed, indicating 109° domain switching.

XRD reciprocal space mapping of Mn-doped $BiFeO_3$ thin film

Figure S2 show X-ray diffraction reciprocal space mapping around BFMO 004 and 114 diffraction spots. In rhombohedral crystal system, {114} diffraction spots of the $(001)_{pc}$-oriented BFMO thin films with 4 different in-plane oriented domains show 3 different spots, because $\bar{1}14$ and $1\bar{1}4$ planes are equivalent. Therefore, single spot in BFMO 004 and 114, as

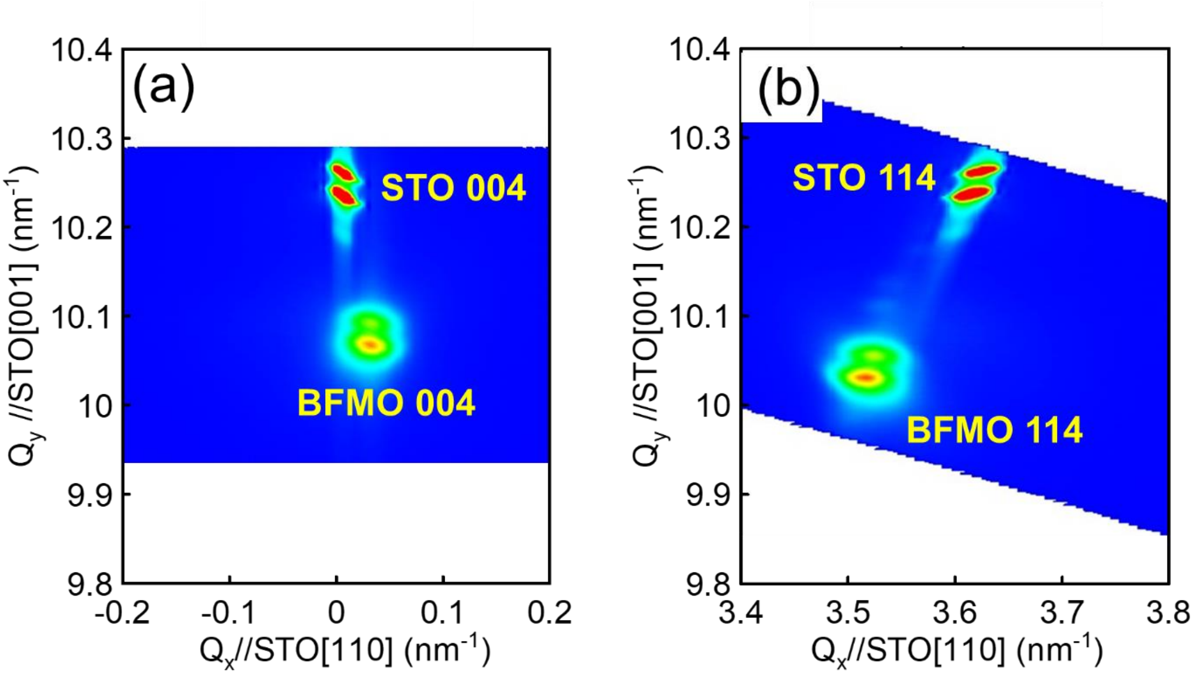

Figure S2. XRD-RSM around (a) BFMO 004 and (b) BFMO 114 diffraction spots.

shown in Fig. S2, means single-domain structure. Double spots of BFO 004 and 114 are due to Cu $K\alpha_1$ and $K\alpha_2$ incidence. The doubling of STO 004 and 114 spots also prove the Cu $K\alpha_1$ and $K\alpha_2$ incidence. This result agreed well with the PFM results in Fig. S1. The angle between $[001]_{pc}$ direction and $(hh0)_{pc}$ plane can be estimated to be 0.63°, meaning the angle between $(hh0)_{pc}$ planes of r_1 and r_3 domains should be 1.26°. This value agreed well with the angle between $(hh0)_{pc}$ planes of r_1 and r_3 domains in intermediate multidomain state of 1.30°.

Sample surface photographs for in-situ normal- and inverse-XFH measurements

Pt electrodes patterns with inter electrodes distances of 10 and 40 μm were used for the sample of in-situ normal- and inverse-XFH measurements under electric field. For applying electric field, Au wires with a diameter of 0.1 mm were bonded on to the Pt electrodes by silver paste, as shown in Fig. S3.

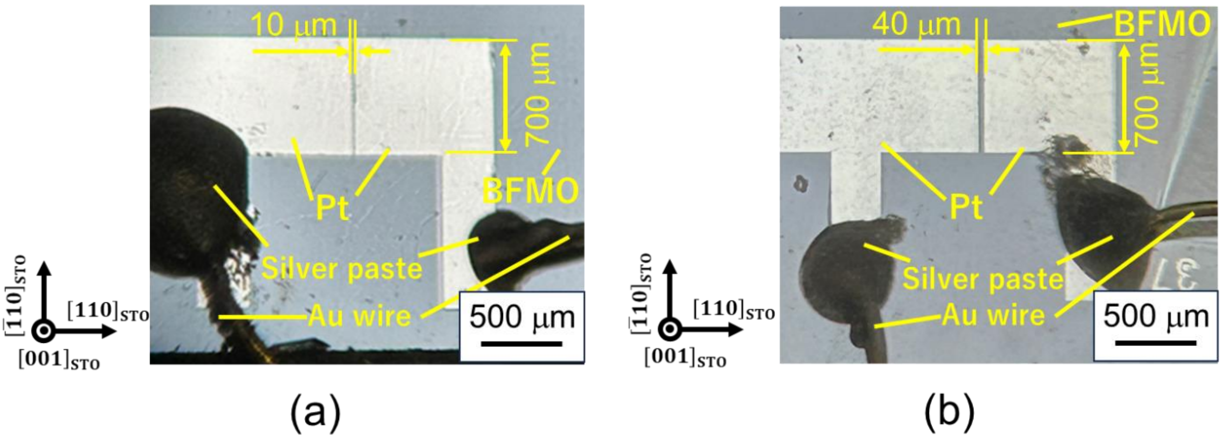

Figure S3. Photographs of the sample surface for in-situ (a) normal- and (b) inverse-XFH measurements under electric field.
